# Correction: Cuban history of CRF19 recombinant subtype of HIV-1

**DOI:** 10.1371/journal.ppat.1010224

**Published:** 2022-01-06

**Authors:** Anna Zhukova, Jakub Voznica, Miraine Dávila Felipe, Thu-Hien To, Lissette Pérez, Yenisleidys Martínez, Yanet Pintos, Melissa Méndez, Olivier Gascuel, Vivian Kouri

There is an error in the affiliations for authors Miraine Dávila Felipe and Olivier Gascuel. Neither author should have a current address listed and both authors have two affiliations.

Both authors are affiliated to #1: Unité Bioinformatique Evolutive, Département de Biologie Computationelle, Institut Pasteur, Paris, France.

The correct second affiliation for Miraine Dávila Felipe is: Université de technologie de Compiègne, LMAC (Laboratory of Applied Mathematics of Compiègne), Compiègne, France.

The correct second affiliation for Olivier Gascuel is: Institut de Systématique, Evolution, Biodiversité (ISYEB—URM 7205 CNRS & Muséum National d’Histoire Naturelle, SU, EPHE & UA), Paris, France.
