# Supplementary material for: Cuban history of CRF19 recombinant subtype of HIV-1
Source: PLoS Pathog. 2021 Aug 9;17(8):e1009786. doi: 10.1371/journal.ppat.1009786 (PMC8376097; doi:10.1371/journal.ppat.1009786)
Supplement: S1 Appendix — The detailed description of the Bayesian analyses performed in this study. (PDF) [file ppat.1009786.s002.pdf]

S1 Appendix. Bayesian analyses. To assess the robustness of the inferences produced in this study, we performed a Bayesian analysis of the CRF19 cluster in BEAST v1.10.4 [1, 2]. We extracted the CRF19 subtree from the *D+CRF19* phylogeny, and pruned the samples that were missing the mode of transmission or sampling date annotations. We have thus obtained a data set of 266 CRF19 sequences. We then performed the dating and ancestral character reconstruction analyses in BEAST on this fixed phylogeny, with an uncorrelated lognormal relaxed molecular clock model [3] (as in Delatorre and Bello's analysis [4]), and Bayesian diffusion in discrete space models [5] for mode of transmission and presence/absence of RT:M41L DRM. MCMC chains were run for 10^8 generations. Adequate chain mixing was assessed by calculating the effective sample size, after excluding an initial 10% using the TRACER v1.7.1 program [6].

BEAST analysis took $2,36$ days on a 96-core machine vs < 1 minute for dating with LSD2 and ACR with PastML on a 1-core machine. It estimated the root date of the CRF19 cluster as 1972, with the 95% HPD interval of $1963 - 1980$. This result is in a perfect agreement with the CRF19 MRCA date estimated by LSD2 on the *D+CRF19* data set: 1974 (CI $1970 - 1977$). However, as expected, the uncertainty is substantially larger as we used more sequences ($D + CRF19$ vs only well-annotated $CRF19$), additional constrains based on diagnostics dates, and a strict molecular clock model for LSD2.

Drug-resistance analysis (see S1 Fig.) detected very similar patterns to those inferred by PastML (Fig 4). Detected TDR clusters were of smaller size (up to 2 patients) due to the fact that we did not include sequences with missing metadata in the Bayesian analysis (e.g. *CU1273_14*, which is part of the largest TDR cluster (5 patients) in the PastML analysis, was not included due to missing transmission mode status).

Transmission mode analysis (see S2 Fig.) however detected different patterns to those inferred by PastML (Fig 3). Bayesian analysis inferred MSM (posterior probability of 0.99) at the origin of the CRF19 epidemics (vs HET with a marginal probability of 0.89 in the PastML analysis). However, the comparison between the two methods is not fully appropriate in this case, as PastML analysis used additional information (risk group at the moment of diagnostics, which was set to the same value as at sampling) to make the predictions. As the states at diagnostics could only be specified on a dated tree, we could not input this information into BEAST. PastML results are in a better agreement with the epidemiological data [7, 8].

References

1. Suchard MA, Lemey P, Baele G, Ayres DL, Drummond AJ, Rambaut A. Bayesian phylogenetic and phylodynamic data integration using BEAST 1.10. Virus Evolution. 2018;4(1). doi:10.1093/ve/vey016.

2. Ayres DL, Darling A, Zwickl DJ, Beerli P, Holder MT, Lewis PO, et al. BEAGLE: An application programming interface and high-performance computing library for statistical phylogenetics. Systematic Biology. 2012;61(1):170–173. doi:10.1093/sysbio/syr100.

3. Drummond AJ, Ho SYW, Phillips MJ, Rambaut A. Relaxed phylogenetics and dating with confidence. PLoS Biol. 2006;4(5):e88.

4. Delatorre E, Bello G. Phylodynamics of the HIV-1 epidemic in Cuba. PloS one. 2013;8(9):e72448. doi:10.1371/journal.pone.0072448.

5. Lemey P, Rambaut A, Drummond AJ, Suchard MA. Bayesian phylogeography finds its roots. PLoS Computational Biology. 2009;5(9):1000520. doi:10.1371/journal.pcbi.1000520.

6. Rambaut A, Drummond AJ, Xie D, Baele G, Suchard MA. Posterior summarization in Bayesian phylogenetics using Tracer 1.7. Systematic Biology. 2018;67(5):901–904. doi:10.1093/sysbio/syy032.

7. Perez Avila J, Pena Torres R, Pena Tores R, Joane Fiol J, Lantero Abreu M, Arazoza Rodriguez H. HIV control in Cuba. Biomedicine and Pharmacotherapy. 1996;50(5):216–219. doi:10.1016/0753-3322(96)87661-X.

8. Miranda Gómez O, Fariñas Reinoso AT, Coutín Marie G, Nápoles Pérez M, Lara Fernández H, Bueno Marrero LE. Panorama of HIV-infection in Cuba, 1986-2007; 2009. 2. Available from: http://scielo.sld.cu/scielo.php?script=sci_arttext&pid=S1561-30032009000200004&lng=es&nrm=iso&tlng=es.
