# Supplementary material for: Cuban history of CRF19 recombinant subtype of HIV-1
Source: PLoS Pathog. 2021 Aug 9;17(8):e1009786. doi: 10.1371/journal.ppat.1009786 (PMC8376097; doi:10.1371/journal.ppat.1009786)
Supplement: S1 Table — Statistics showing the numbers of sequences and prevalence of the SDRMs that were not present in treatment-naive CRF19 sequences used in this study. [file ppat.1009786.s001.pdf]

S1 Table. Statistics on SDRMs not present in treatment-naive CRF19 sequences.

SDRM	sequences with SDRM	prevalence
RT:K219E	19	6.07%
RT:T215F	19	6.07%
RT:K219Q	16	5.11%
PR:I84V	8	2.56%
RT:K65R	8	2.56%
PR:L24I	7	2.24%
RT:T215I	7	2.24%
PR:M46L	6	1.92%
RT:L100I	6	1.92%
PR:F53L	5	1.60%
PR:I54L	5	1.60%
PR:I54M	5	1.60%
PR:V32I	5	1.60%
RT:T69D	5	1.60%
PR:G48V	3	0.96%
PR:G73S	3	0.96%
RT:M230L	3	0.96%
RT:P225H	3	0.96%
RT:V106A	3	0.96%
RT:Y115F	3	0.96%
PR:I50V	2	0.64%
PR:V82T	2	0.64%
RT:K103S	2	0.64%
RT:K219R	2	0.64%
RT:T215V	2	0.64%
RT:V106M	2	0.64%
RT:Y181I	2	0.64%
PR:G73C	1	0.32%
PR:I85V	1	0.32%
PR:L23I	1	0.32%
PR:L76V	1	0.32%
PR:N83D	1	0.32%
RT:D67E	1	0.32%
RT:G190E	1	0.32%
RT:K101P	1	0.32%
RT:V75T	1	0.32%
RT:Y181V	1	0.32%
